# Arsenic Impairs Critical Cellular Events of Human Cardiac Development Across Differentiation and Metabolic Maturation

**DOI:** 10.64898/2026.10.11.758344

**Published:** 2026-10-12

**Authors:** Bailey Renee Skeen, Cate Duncan, Fanrong Yao, Brian Shewchuk, Karen Mruk, Erik J. Tokar, Xian Wu

## Abstract

Arsenic (As) is a widespread environmental contaminant, with exposure occurring primarily through contaminated food and water or cigarette smoke. Maternal exposure to As represents a significant risk factor for congenital heart disease in offspring. However, the mechanisms underlying As-induced developmental cardiotoxicity remain poorly defined. Pluripotent stem cells provide an excellent platform for studying mechanisms of developmental toxicity. Embryoid bodies (EBs) derived from stem cells, can recapitulate many key events of early embryogenesis including germ layers specification. Human cardiomyocytes are derived from mesodermal progenitors. NKX2- 5 governs cardiac specification and morphogenesis, and its mutation produces atrial and ventricular defects in humans. Using an NKX2-5 reporter stem cell line, we modeled mesoderm differentiation in EBs and cardiac induction in a 2D cardiomyocyte differentiation system. In an 8-day differentiation protocol, As (0.05 μM; 3.75 ppb) significantly suppressed cardiomyocyte differentiation and cardiac induction. However, As increased mesodermal markers (HAND1, SNAI2, HOPX) gene expression. In contrast, cardiac-specific genes, including NKX2-5, GATA4, Troponin T, and α-Actinin, were markedly downregulated in the presence of As. As also reduced expression of genes related to cardiac induction, fibroblast function, and vascular markers in the cardiac organoid model. As impaired cardiomyocyte maturation related gene expressions at concentrations without affecting cardiomyocyte proliferation. In a zebrafish model of early heart development, As increased heart rate and disrupted ion channel gene expression. In conclusion, As inhibits ESC differentiation into cardiomyocytes and disrupts cardiomyocyte maturation. Combining stem cell–derived models with zebrafish exposure, this study illuminates adverse outcome pathways for As-induced congenital heart defect.

